# Global, regional, and national burdens of interpersonal violence in young women aged 10–24 years from 1990 to 2019: a trend analysis based on the global burden of disease study 2019

**DOI:** 10.3389/fpsyg.2023.1241862

**Published:** 2024-01-11

**Authors:** Yu Cao, Hao Lu, Pengqian Duan, Dongmei Wang, Guojun Wei

**Affiliations:** ^1^The Fourth Affiliated Hospital Zhejiang University School of Medicine, Yiwu, Zhejiang Province, China; ^2^School of Medicine, Xiamen University, Xiamen, Fujian Province, China; ^3^Xiang'an Hospital of Xiamen University, Xiamen, Fujian Province, China

**Keywords:** GBD (global burden of disease), interpersonal violence, young women, jointpoint regression, sexual violence

## Abstract

**Background:**

Interpersonal violence (IPV) against young women, including physical and sexual violence, poses a major threat to public health. We analyzed global, regional and national trends in violence against females aged 10–24 years from 1990 to 2019.

**Methods:**

We extracted age-standardized prevalence rates (ASPRs) of physical violence by firearm (PVF), physical violence by other means (PVOM), physical violence by sharp object (PVSO), and sexual violence (SV) from the Global Burden of Diseases, Injuries, and Risk Factors Study 2019. Joinpoint regression analysis calculated annual and average annual percentage changes (AAPCs) in ASPRs.

**Results:**

Globally, the ASPRs of the four measures of IPV decreased between 1990 and 2019, with the steepest declines between 2000 and 2009, except for SV, which increased slightly. However, the ASPRs of PVF and PVOM increased slightly between 2010 and 2019. Regionally, PVF prevalence declined most in East Asia (−0.9505, −1.0011 to −0.8975), South Asia (−0.277, −0.3089 to −0.244) and Latin America but PVOM prevalence increased in Oceania (0.6275, 0.6036 to 0.6498) and SV prevalence increased in Caribbean (0.4267, 0.4069 to 0.4495). Nationally, PVF prevalence decreased most in Thailand (−2.4031, −2.4634 to −2.3328) but increased most in Libya (6.8143, 6.6194 to 7.0113). SV prevalence increased most in Oman (0.4561, 0.4338 to 0.478) and the largest increase in Disability-adjusted life years (DALYs) from PVOM was observed in Botswana (6.2725, 6.0951 to 6.4082). DALYs showed similar trends.

**Conclusion:**

While global declines over 30 years are encouraging, IPV against young women persists. Urgent, tailored approaches across sectors are critical to curb drivers of violence against young women, including poverty, inequality and sociocultural attitudes. High-quality data and in-depth analyses can inform locally-relevant solutions. Overall, intensified political will and resource investment are needed to overcome this pervasive human rights violation.

## Introduction

In 2019, interpersonal violence (IPV) constituted the fifth leading contributor to disability-adjusted life-years (DALYs) among adolescents and young adults within the age cohort of 10 to 24 years (GBD Diseases and Injuries Collaborators, 2020). Violence targeted against women is now widely acknowledged as a public health issue and human rights transgression bearing global ramifications (Krantz, 2002). The umbrella expression of “violence against women” encapsulates myriad forms of maltreatment perpetrated against women and girls over the course of their lifespans (Krantz and Garcia-Moreno, 2005). Violence can manifest itself in physical, sexual, or psychological forms. A preceding investigation, employing a population-based national sample, appraised that 60% of women have endured at least one typology of violence during their adult lifespan (Moracco et al., 2007).

IPV remains prevalent due in part to widespread myths and stereotypes that wrongly blame victims and minimize the severity of abuse (Bannon and Salwen-Deremer, 2018; Leung, 2019; Lilley et al., 2023). Exposure to IPV has profound adverse effects on mental and physical health, including increased risks for depression, PTSD, suicidality, somatic disorders, and poor reproductive health outcomes (Lagdon et al., 2014; Stewart et al., 2016; Conroy et al., 2023; Debowska et al., 2023; Sharratt et al., 2023). Effective solutions must address attitudes and beliefs accepting of IPV in addition to developing interventions and support for survivors (Smith et al., 2020; Conroy et al., 2023; Hudspith et al., 2023).

At the current juncture, the prevalence of IPV has been scrutinized in circumscribed geographical expanses, predominantly in discrete regions or particular nations based on data gleaned from cohorts or registry mechanisms (Leone et al., 2019; Sanawar et al., 2019; Ribeiro et al., 2021; van Daalen et al., 2022). While preceding studies have provided invaluable data, additional cross-continental and cross-national comparisons have been impeded by methodological heterogeneities in prevalence estimation across discrete analyses. Consequently, a comprehensive global perspective on IPV prevalence remains obscure. Undoubtedly, a rigorous and systematic assessment of the magnitude and extent of IPV is of profound relevance and significance for cultivating an enhanced understanding of the epidemiology of these conditions and for informing public health policy development.

The Global Burden of Diseases, Injuries, and Risk Factors Study (GBD) 2019 (Institute of Health Metrics and Evaluation, n.d.) provides an invaluable data repository to enable rigorous epidemiological inquiry and analysis. The expansive data compiled within the GBD study can be exploited to delineate disease burden and evaluate epidemiological trends. Specifically, these data can be used to quantify the current state as well as temporal changes in disease burden across regions and countries. By illuminating disease dynamics geographically and over time, analyses of GBD data may provide critical insights to guide evidence-based clinical practice and health policy decision making (Paik et al., 2022). We utilized data aggregated at the global scale in the GBD 2019 study to describe the contemporary epidemiological landscape in 2019 and analyze longitudinal variations in incidence, DALYs and prevalence attributable to IPV including Physical violence by firearm (PVF), Physical violence by other means (PVOM), Physical violence by sharp object (PVSO) and Sexual violence (SV) by decade since 1990. We then identified the year exhibiting the most significant changes in trends for the aforementioned metrics. Global trends were stratified by age group and sociodemographic index (SDI). Trends at regional and national levels were also reported.

## Methods

### Study design and data sources

In the analysis of GBD 2019 data (accessible at https://vizhub.healthdata.org/gbd-results/), we acquired recurrent cross-sectional data pertaining to the worldwide prevalence of PVF, PVOM, PVSO, and SV, sourced from the Global Health Data Exchange (GHDx) (GBD Diseases and Injuries Collaborators, 2020). The GHDx includes data on incidence, prevalence, mortality, and DALYs for 369 diseases in 204 countries from 1990 to 2019 (GBD Diseases and Injuries Collaborators, 2020). Data were collected for women across three distinct age groups (10–14 years, 15–19 years, and 20–24 years), and for the 21 regional country groupings delineated within the framework of the GBD project (GBD Diseases and Injuries Collaborators, 2020). The primary objective of the GBD regional groupings is to optimize epidemiological homogeneity across different regions (GBD Demographics Collaborators, 2020).

The GBD 2019 additionally computed a SDI for each nation, serving as a composite metric reflecting the interrelation between social and economic development and health outcomes. Integrates lag-distributed income *per capita*, mean educational attainment, and the total fertility rate (GBD Diseases and Injuries Collaborators, 2020; GBD Risk Factors Collaborators, 2020). The SDI is divided into five quintiles, encompassing categories of low, low-middle, middle, high-middle, and high levels of development (GBD Diseases and Injuries Collaborators, 2020).

Incident cases, prevalent cases, DALYs, incidence rates, prevalence rates, DALYs rates and mortality for PVF, PVOM, PVSO, and SV were directly extracted from the GBD 2019 dataset (if available) (GBD Diseases and Injuries Collaborators, 2020). Crude rates are reported per 100,000 population. The 95% uncertainty intervals (UIs) were delineated by the 25th and 975th values among the 1,000 estimates derived through the GBD modeling methodology (Foreman et al., 2012; GBD Diseases and Injuries Collaborators, 2020).

The GBD methodology employs diverse measurement tools to assess non-fatal disease burden, including DisMod-MR modeling for epidemiological data and disability weights derived from pairwise comparisons between health states (Salomon et al., 2012, 2015; GBD Diseases and Injuries Collaborators, 2020). DisMod-MR 2.1 constitutes a Bayesian meta-regression instrument employed for the synthesis of sparse and heterogeneous data related to prevalence, incidence, remission and mortality (Flaxman et al., 2015). Disability weights measure the severity of health loss arising from non-fatal outcomes, utilizing a scale ranging from 0 (representing full health) to 1 (indicating death) (Salomon et al., 2012).

### Statistical analysis

Joinpoint regression analysis was employed to interrogate temporal trends in PVF, PVOM, PVSO and SV prevalence at global, continental, and national levels. The analysis identified points of significant change in trend (joinpoints) and partitioned the composite temporal trend into constituent segments demarcated by identified joinpoints. The epidemiological trajectory of each segment was further evaluated through the computation of the annual percentage change (APC) and its corresponding 95% confidence interval (CI). Additionally, the weighted average APC (AAPC) over four periods (1990–1999, 2000–2009, 2010–2019 and 1990–2019), where the weights were determined by the interval width of each segment, was also calculated (Clegg et al., 2009). The final model in the Joinpoint software was selected based on a synthesis of the authors’ professional insights and the application of Data Driven Weighted Bayesian Information Criterion (WBIC) methods. If the point estimate of the APC/AAPC, along with the lower bound of its 95% CI, both surpass zero, it indicates a discernible upward trend within the designated temporal span. Alternatively, in the event that both the point estimate of the APC/AAPC and the upper bound of its 95% CI fall below zero, an observed diminishing trend within the designated temporal span was identified. Otherwise, the presence of a consistent trend within the specified temporal interval was detected.

Under the auspices of the University of Washington’s research ethics board, the GBD study adheres rigorously to an approved protocol. The funding entity played no part in the design, analysis, interpretation, or publication of this study. Statistical analyses and graphical visualizations were conducted utilizing R software (version 4.2.1) for all computations.

## Results

### Global trends

Globally, the prevalence of PVF, PVOM, PVSO and SV among young women declined between 1990 and 2019 [Annual average percentage change (AAPC) -0.62 (95% CI -0.67 to −0.57), −0.57 (−0.6 to −0.54), −0.97 (−0.99 to −0.96), −0.47 (−0.48 to −0.46), respectively] (Table 1).

**Table 1:** Global AAPCs in prevalence, incidence, Deaths, and DALYs of PVF, PVOM, PVSO and SV.

	DALYs	Deaths	Incidence	Prevalence
	AAPC (95% CI)	AAPC (95% CI)	AAPC (95% CI)	AAPC (95% CI)
**Physical violence by firearm**				
1990–1999	1.11 (0.66 to 1.68)*	1.13 (0.7 to 1.67)*	0.55 (0.48 to 0.62)*	−1.17 (−1.27 to −1.07)*	
2000–2009	−2.44 (−3.31 to −1.79)*	−2.45 (−3.33 to −1.81)*	−0.07 (−0.11 to −0.02)*	−1.77 (−1.86 to −1.68)*	
2010–2019	−0.56 (−1.5 to −0.08)*	−0.67 (−1.26 to −0.29)*	0.68 (0.47 to 0.89)*	0.96 (0.7 to 1.21)*	
1990–2019	−0.35 (−0.52 to −0.16)*	−0.34 (−0.52 to −0.14)*	0.35 (0.3 to 0.4)*	−0.62 (−0.67 to −0.57)*	
*Physical violence by other means*				
1990–1999	0.12 (−0.34 to 0.59)	0.26 (−0.23 to 0.78)	−0.75 (−0.78 to −0.72)*	−1.08 (−1.12 to −1.04)*	
2000–2009	−2.41 (−2.6 to −2.2)*	−2.46 (−2.7 to −2.21)*	−0.83 (−0.86 to −0.81)*	−1.23 (−1.27 to −1.19)*	
2010–2019	−1.67 (−1.97 to −1.45)*	−1.93 (−2.21 to −1.73)*	−0.06 (−0.09 to −0.03)*	0.66 (0.44 to 0.87)*	
1990–2019	−1.3 (−1.38 to −1.22)*	−1.32 (−1.41 to −1.24)*	−0.56 (−0.57 to −0.56)*	−0.57 (−0.6 to −0.54)*	
*Physical violence by sharp object*				
1990–1999	0.03 (−0.55 to 0.6)	0.09 (−0.51 to 0.69)	−1.26 (−1.33 to −1.18)*	−1.33 (−1.37 to −1.26)*	
2000–2009	−3.35 (−3.75 to −2.99)*	−3.38 (−3.81 to −2.98)*	−1.26 (−1.31 to −1.2)*	−1.75 (−1.79 to −1.7)*	
2010–2019	−1.75 (−2.11 to −1.45)*	−1.86 (−2.24 to −1.55)*	−0.16 (−0.25 to −0.09)*	0.18 (0.14 to 0.22)*	
1990–2019	−1.58 (−1.71 to −1.45)*	−1.59 (−1.73 to −1.45)*	−0.92 (−0.94 to −0.9)*	−0.97 (−0.99 to −0.96)*	
*Sexual violence*				
1990–1999	−0.61 (−0.67 to −0.57)*	NA	NA	−0.59 (−0.64 to −0.54)*	
2000–2009	0.52 (0.45 to 0.58)*	NA	NA	0.51 (0.45 to 0.57)*	
2010–2019	−1.47 (−1.56 to −1.39)*	NA	NA	−1.41 (−1.48 to −1.35)*	
1990–2019	−0.49 (−0.51 to −0.48)*	NA	NA	−0.47 (−0.48 to −0.46)*	

*Indicates that the data is significantly different from zero at alpha = 0.05 level. AAPC, average annual percentage change. DALYs, disability-adjusted life-years.

The steepest decreases were observed between 2000 and 2009 for PVF, PVOM and PVSO [AAPC -1.77 (−1.86 to −1.68), −1.23 (−1.27 to −1.19), −1.75 (−1.79 to −1.7)], while SV prevalence increased slightly during this period [AAPC 0.51 (0.45 to 0.57)]. Between 2010 and 2019, the prevalence of PVF and PVOM increased slightly [AAPC 0.96 (0.7 to 1.21), 0.66 (0.44 to 0.87)] and PVSO stabilized [AAPC 0.18 (0.14 to 0.22)], while SV prevalence declined substantially [AAPC -1.41 (−1.48 to −1.35)]. The PVF, PVOM, PVSO, SV DALYs and Deaths also decreased between 1990 and 2019, and declined much more between 2000 and 2009. However, in Incidence’s results, PVF increased a little between 1990 and 2019, while PVOM, PVSO decreased in the same period (Table 1).

**Figure 1:**
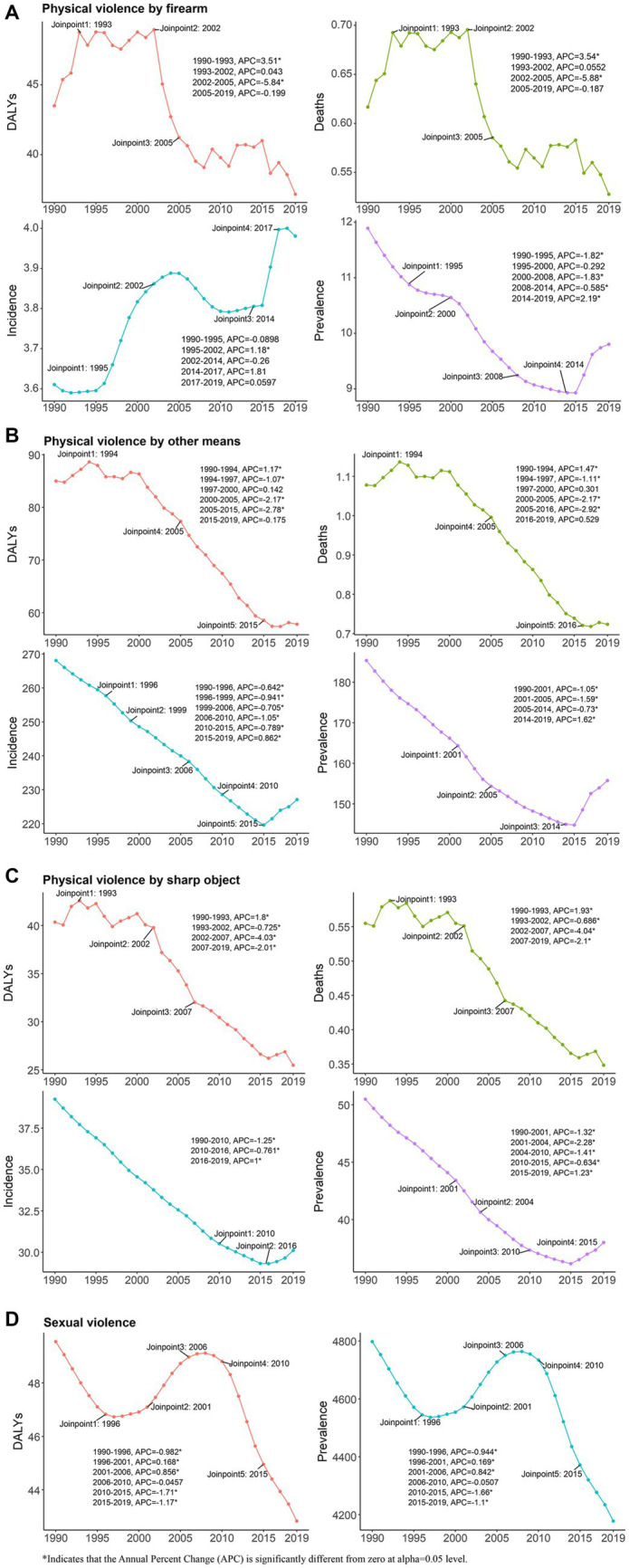
Joinpoint regression analysis of global Physical violence by firearm **(A)**, Physical violence by other means **(B)**, Physical violence by sharp object **(C)** and Sexual violence **(D)** prevalence, incidence, Deaths, and DALYs in young women aged 10–24 years from 1990 to 2019. DALYs, Disability-adjusted life years.

In summary, despite some fluctuations over time, the global prevalence of the four measures of violence against women decreased between 1990 and 2019. Joinpoint regression analysis identified turning points in the prevalence trends for each measure around 1995–2015 (Figure 1).

### Global trends by age group

Globally, the largest decrease in PVF prevalence was in 15-19-year-olds, from 11.87 (8.51–15.91) in 1990 to 9.6 (7.13–12.87) in 2019 [AAPC -0.67, (−0.71 to −0.62)] (Table 2). PVOM prevalence decreased in all groups between 1990 and 2019, with little difference in decrease between 10–14-year-olds and 20-24-year-olds [AAPC -0.6, (−0.65 to −0.54); −0.6, (−0.62 to −0.58)] (Table 2). SV prevalence decreased between 1990 and 2019 in all groups (Table 2), with little difference between 15-19-year-olds and 20-24-year-olds compared to PVF and PVOM, but the lowest decrease was in 10–14-year-olds [AAPC -0.27, (−0.28 to −0.26)].

**Table 2:** The prevalence and DALYs of PVF, PVOM, PVSO and SV and their AAPCs from 1990 to 2019 at the global and regional levels.

	Prevalence					DALYs				
	Cases (n), 1990	Prevalence (per 100,000 population), 1990	Cases (n), 2019	Prevalence (per 100,000 population), 2019	AAPC, 1990–2019	Cases (n), 1990	DALYs (per 100,000 population), 1990	Cases (n), 2019	DALYs (per 100,000 population), 2019	AAPC, 1990–2019
*Physical violence by firearm*											
Global	90583.32 (65640.66 to 119710.66)	11.89 (8.62 to 15.71)	89038.04 (66992.55 to 115,303)	9.8 (7.37 to 12.69)	−0.62 (−0.67 to −0.57)*	331394.58 (314625.74 to 350653.35)	43.5 (41.3 to 46.03)	337587.43 (302886.15 to 376151.59)	37.16 (33.34 to 41.41)	−0.35 (−0.52 to −0.16)*	
Age group, years											
10–14 years	22141.22 (15220.69 to 30921.68)	8.45 (5.81 to 11.81)	21698.92 (15610.79 to 29039.78)	6.98 (5.02 to 9.34)	−0.61 (−0.67 to −0.55)*	41,905 (38793.23 to 45419.76)	16 (14.81 to 17.34)	40714.06 (35991.1 to 46019.95)	13.1 (11.58 to 14.8)	−0.7 (−0.82 to −0.57)*	
15–19 years	30343.79 (21735.96 to 40646.22)	11.87 (8.51 to 15.91)	28979.17 (21503.27 to 38847.71)	9.6 (7.13 to 12.87)	−0.67 (−0.71 to −0.62)*	135005.1 (126234.49 to 145076.51)	52.83 (49.4 to 56.78)	134813.58 (120616.65 to 150370.61)	44.68 (39.97 to 49.83)	−0.53 (−0.76 to −0.35)*	
20–24 years	38098.32 (28398.81 to 49,429)	15.59 (11.62 to 20.23)	38359.95 (29570.46 to 49597.93)	12.97 (10 to 16.77)	−0.6 (−0.65 to −0.57)*	154484.48 (145145.88 to 166098.54)	63.22 (59.4 to 67.97)	162059.8 (144228.94 to 181659.95)	54.79 (48.76 to 61.42)	−0.47 (−0.62 to −0.33)*	
Sociodemographic index											
High-middle SDI	24363.48 (17220.41 to 32986.61)	16.41 (11.6 to 22.22)	17229.93 (12970.3 to 22411.14)	14.17 (10.66 to 18.43)	−0.47 (−0.5 to −0.43)*	40702.52 (38377.94 to 43369.62)	27.42 (25.85 to 29.22)	26422.39 (24335.71 to 28704.76)	21.72 (20.01 to 23.6)	−0.62 (−0.91 to −0.15)*	
High SDI	17571.09 (13391.34 to 23113.72)	19.66 (14.99 to 25.87)	17347.46 (13181.04 to 22566.38)	20.43 (15.52 to 26.57)	0.14 (0.09 to 0.18)*	59538.9 (57994.05 to 61206.6)	66.63 (64.9 to 68.49)	37854.83 (36202.58 to 39733.44)	44.58 (42.63 to 46.79)	−1.2 (−1.55 to −0.86)*	
Low-middle SDI	7510.09 (5545.94 to 9908.48)	4.31 (3.18 to 5.69)	14753.69 (11270.44 to 19073.23)	5.88 (4.49 to 7.61)	1.13 (1.08 to 1.17)*	64703.03 (58409.51 to 70917.88)	37.14 (33.53 to 40.71)	110564.12 (94260.81 to 129037.26)	44.09 (37.59 to 51.46)	0.29 (−0.19 to 0.61)	
Low SDI	2382.68 (1697.2 to 3269.11)	2.94 (2.09 to 4.03)	8107.14 (5743.55 to 11321.77)	4.39 (3.11 to 6.14)	1.45 (1.4 to 1.49)*	29075.03 (23100.32 to 35295.2)	35.84 (28.48 to 43.51)	54611.36 (39765.09 to 71157.22)	29.61 (21.56 to 38.57)	−0.6 (−0.81 to −0.37)*	
Middle SDI	38718.84 (27482.82 to 52439.73)	14.43 (10.24 to 19.55)	31541.98 (23618.47 to 41046.08)	11.86 (8.88 to 15.43)	−0.65 (−0.72 to −0.58)*	137056.35 (126,800 to 149456.53)	51.09 (47.27 to 55.71)	107771.08 (96206.46 to 121167.18)	40.51 (36.16 to 45.54)	−0.95 (−1.12 to −0.76)*	
Region											
Andean Latin America	315.24 (244.75 to 408.92)	5.1 (3.96 to 6.61)	864.88 (676.47 to 1111.45)	10.6 (8.29 to 13.62)	2.62 (2.57 to 2.66)*	2658.96 (2020.15 to 3409.19)	42.99 (32.67 to 55.13)	2949.67 (2125.88 to 3904.41)	36.14 (26.04 to 47.83)	−0.69 (−0.88 to −0.5)*	
Australasia	297.36 (236.32 to 378.02)	12.59 (10 to 16)	414.29 (324.51 to 528.33)	15.61 (12.23 to 19.91)	0.77 (0.71 to 0.82)*	591.2 (505.85 to 698.44)	25.02 (21.41 to 29.56)	157.76 (130.74 to 191.51)	5.94 (4.93 to 7.22)	−5.17 (−5.96 to −4.4)*	
Caribbean	750.33 (579.14 to 974.55)	14.01 (10.81 to 18.19)	1042.31 (802.42 to 1349.42)	18.35 (14.13 to 23.76)	0.98 (0.95 to 1.01)*	6882.21 (5418.43 to 9031.49)	128.46 (101.14 to 168.58)	8257.07 (5301.93 to 12148.07)	145.41 (93.37 to 213.93)	0.35 (0.15 to 0.5)*	
Central Asia	264.92 (211.36 to 330.69)	2.69 (2.15 to 3.36)	459.86 (366.14 to 576.15)	4.18 (3.33 to 5.23)	1.58 (1.51 to 1.64)*	677.27 (609.05 to 760.7)	6.89 (6.19 to 7.73)	293.74 (253.48 to 336.77)	2.67 (2.3 to 3.06)	−3.18 (−3.63 to −2.68)*	
Central Europe	981.75 (778.75 to 1256.09)	6.99 (5.54 to 8.94)	1022.71 (800.95 to 1306.99)	11.71 (9.17 to 14.97)	1.82 (1.74 to 1.87)*	1830.31 (1580.27 to 2117.38)	13.02 (11.24 to 15.07)	680.84 (563.24 to 826.6)	7.8 (6.45 to 9.47)	−2.04 (−2.46 to −1.65)*	
Central Europe, Eastern Europe, and Central Asia	2760.13 (2198.34 to 3492.7)	5.85 (4.66 to 7.41)	2572.06 (2048.44 to 3220.71)	7.24 (5.77 to 9.07)	0.76 (0.68 to 0.82)*	5980.93 (5655.88 to 6308.75)	12.68 (11.99 to 13.38)	2115.15 (1887.35 to 2360.78)	5.96 (5.32 to 6.65)	−2.71 (−3.25 to −2.14)*	
Central Latin America	7805.98 (5881.03 to 10108.6)	28.52 (21.49 to 36.93)	13167.49 (9888.33 to 17061.39)	40.33 (30.29 to 52.26)	1.29 (1.18 to 1.38)*	72874.43 (69351.56 to 76767.01)	266.23 (253.36 to 280.45)	98547.1 (81475.65 to 118628.04)	301.86 (249.57 to 363.38)	0.1 (−0.91 to 0.65)	
Central Sub-Saharan Africa	126.38 (98.69 to 159.56)	1.46 (1.14 to 1.85)	491.18 (380.44 to 617.24)	2.28 (1.77 to 2.87)	1.56 (1.54 to 1.58)*	2197.37 (1349.23 to 3281.3)	25.45 (15.63 to 38.01)	4717.71 (2913.8 to 7012.97)	21.94 (13.55 to 32.61)	−0.35 (−0.51 to −0.2)*	
East Asia	43158.01 (29162.7 to 61720.23)	23.72 (16.03 to 33.93)	20172.6 (14566.97 to 26986.36)	18.18 (13.13 to 24.32)	−0.95 (−1 to −0.9)*	4883.45 (3942.79 to 5969.09)	2.68 (2.17 to 3.28)	1598.3 (1302.75 to 1932.74)	1.44 (1.17 to 1.74)	−2.18 (−2.31 to −2.02)*	
Eastern Europe	1513.46 (1200.35 to 1913.09)	6.5 (5.16 to 8.22)	1089.49 (873.48 to 1374.64)	6.91 (5.54 to 8.72)	0.28 (0.18 to 0.35)*	3473.35 (3304.46 to 3625.1)	14.92 (14.2 to 15.58)	1140.56 (981.3 to 1315.62)	7.23 (6.22 to 8.34)	−2.7 (−4.11 to −1.51)*	
Eastern Sub-Saharan Africa	1148.87 (818.17 to 1589.51)	3.68 (2.62 to 5.09)	3586.54 (2527.3 to 5001.39)	5.13 (3.61 to 7.15)	1.23 (1.14 to 1.31)*	15262.44 (11188.77 to 20,219)	48.87 (35.82 to 64.73)	28358.73 (20518.57 to 38127.2)	40.52 (29.32 to 54.48)	−0.66 (−0.72 to −0.6)*	
High-income	18349.4 (14190.75 to 24081.9)	18.43 (14.25 to 24.19)	17744.96 (13599.76 to 23022.78)	19.16 (14.68 to 24.85)	0.14 (0.09 to 0.18)*	65038.77 (63422.45 to 66904.87)	65.33 (63.71 to 67.21)	42350.4 (40531.2 to 44287.31)	45.72 (43.76 to 47.81)	−1.03 (−1.35 to −0.72)*	
High-income Asia Pacific	1929.54 (1466.82 to 2475.81)	9.4 (7.14 to 12.06)	1694.94 (1287.04 to 2174.3)	13 (9.87 to 16.68)	1.15 (1.05 to 1.23)*	328.81 (289.76 to 375.95)	1.6 (1.41 to 1.83)	173.49 (142.09 to 207.45)	1.33 (1.09 to 1.59)	−0.59 (−0.82 to −0.35)*	
High-income North America	10630.09 (7986.66 to 14641.16)	35.67 (26.8 to 49.13)	9989.68 (7459.48 to 13434.02)	28.78 (21.49 to 38.7)	−0.73 (−0.77 to −0.69)*	54112.63 (52672.01 to 55756.39)	181.59 (176.75 to 187.1)	35829.04 (34277.89 to 37623.97)	103.22 (98.75 to 108.39)	−1.94 (−2.3 to −1.65)*	
Latin America and Caribbean	14959.35 (11020.87 to 19744.28)	23.78 (17.52 to 31.39)	22533.99 (16846.27 to 29146.06)	31.19 (23.31 to 40.34)	1.01 (0.91 to 1.09)*	130343.37 (124648.55 to 136283.78)	207.21 (198.16 to 216.65)	176868.66 (156497.58 to 200370.73)	244.78 (216.58 to 277.3)	0.49 (−0.02 to 0.81)	
North Africa and Middle East	2344.56 (1831.05 to 3008.72)	4.35 (3.4 to 5.58)	6849.85 (5373.47 to 8787.36)	8.78 (6.89 to 11.26)	2.45 (2.43 to 2.47)*	11949.76 (10185.44 to 14368.96)	22.17 (18.9 to 26.66)	18312.24 (14188.03 to 23553.07)	23.47 (18.19 to 30.19)	0.69 (−0.25 to 1.56)	
Oceania	23.67 (18.81 to 29.73)	2.37 (1.88 to 2.98)	66.27 (53.35 to 82.93)	3.56 (2.87 to 4.46)	1.45 (1.42 to 1.48)*	107.39 (73.5 to 153.87)	10.75 (7.36 to 15.41)	437.13 (281.27 to 651.82)	23.5 (15.12 to 35.04)	2.77 (2.19 to 3.38)*	
South Asia	1288.28 (1012.64 to 1615.3)	0.8 (0.63 to 1)	2600.05 (2061.59 to 3230.08)	1.02 (0.81 to 1.27)	0.87 (0.82 to 0.92)*	24683.49 (19841.1 to 30152.74)	15.27 (12.28 to 18.66)	17382.95 (13395.45 to 22191.24)	6.83 (5.26 to 8.72)	−2.86 (−3.1 to −2.6)*	
Southeast Asia	3291.6 (2650.13 to 4129.69)	4.44 (3.58 to 5.58)	4213.12 (3308.59 to 5361.31)	5.06 (3.97 to 6.43)	0.44 (0.37 to 0.49)*	43621.01 (35,044 to 55037.7)	58.89 (47.31 to 74.3)	14622.71 (11966.95 to 17618.07)	17.55 (14.36 to 21.15)	−3.99 (−4.25 to −3.74)*	
Southeast Asia, East Asia, and Oceania	46473.28 (31970.73 to 65,467)	18.08 (12.44 to 25.47)	24,452 (17971.44 to 32264.34)	12.47 (9.16 to 16.45)	−1.28 (−1.32 to −1.25)*	48611.85 (39775.57 to 60217.4)	18.92 (15.48 to 23.43)	16658.14 (13900.8 to 19644.82)	8.49 (7.09 to 10.02)	−2.73 (−3.05 to −2.45)*	
Southern Latin America	1059.6 (809.1 to 1389.41)	16.02 (12.23 to 21)	1376.8 (1072.63 to 1765.44)	18.33 (14.28 to 23.51)	0.5 (0.47 to 0.54)*	4590.53 (4099.85 to 5133.36)	69.39 (61.97 to 77.59)	4223.08 (3552.42 to 5014.62)	56.23 (47.3 to 66.77)	−0.65 (−1.1 to −0.12)*	
Southern Sub-Saharan Africa	884.87 (624.17 to 1236.24)	9.96 (7.02 to 13.91)	953.13 (666.19 to 1352.84)	8.99 (6.28 to 12.76)	−0.33 (−0.41 to −0.27)*	15366.43 (12515.86 to 19034.79)	172.93 (140.85 to 214.21)	3959.62 (2536.73 to 5747.09)	37.34 (23.92 to 54.2)	−5.18 (−5.36 to −5.03)*	
Sub-Saharan Africa	4408.33 (3051.69 to 6294.38)	5.53 (3.83 to 7.9)	12285.14 (8476.88 to 17691.63)	6.85 (4.73 to 9.87)	0.79 (0.74 to 0.84)*	44786.42 (37076.52 to 53038.41)	56.19 (46.52 to 66.55)	63899.89 (47044.55 to 84240.14)	35.64 (26.24 to 46.99)	−1.61 (−1.8 to −1.41)*	
Tropical Latin America	6087.8 (4318.79 to 8193.89)	25.38 (18 to 34.16)	7459.31 (5426.68 to 9866.97)	28.95 (21.06 to 38.29)	0.51 (0.44 to 0.56)*	47927.76 (45617.09 to 50691.9)	199.78 (190.15 to 211.3)	67114.82 (62871.13 to 71704.48)	260.44 (243.97 to 278.25)	0.91 (0.69 to 1.15)*	
Western Europe	4432.81 (3516.73 to 5607.67)	11.02 (8.74 to 13.94)	4269.23 (3350.29 to 5425.99)	12.3 (9.65 to 15.63)	0.39 (0.31 to 0.46)*	5415.6 (5118.39 to 5703.15)	13.46 (12.72 to 14.17)	1967.03 (1803.18 to 2152.65)	5.67 (5.19 to 6.2)	−3.07 (−3.46 to −2.71)*	
Western Sub-Saharan Africa	2248.2 (1503.7 to 3372.61)	7.26 (4.86 to 10.9)	7254.29 (4852.02 to 10803.76)	9.4 (6.29 to 14)	0.94 (0.9 to 0.97)*	11960.19 (9146.62 to 15417.6)	38.65 (29.55 to 49.82)	26863.83 (18597.7 to 39410.97)	34.8 (24.09 to 51.05)	−0.84 (−1.24 to −0.48)*	
*Physical violence by other means*											
Global	1411939.44 (1071562.08 to 1815693.07)	185.34 (140.66 to 238.34)	1414741.91 (1058088.58 to 1832644.07)	155.74 (116.48 to 201.75)	−0.57 (−0.6 to −0.54)*	647477.08 (588044.67 to 711727.83)	84.99 (77.19 to 93.43)	525236.83 (462249.25 to 593375.3)	57.82 (50.89 to 65.32)	−1.3 (−1.38 to −1.22)*	
Age group, years											
10–14 years	305041.8 (227177.37 to 400755.45)	116.46 (86.74 to 153.01)	299519.61 (220730.13 to 397167.6)	96.35 (71.01 to 127.77)	−0.6 (−0.65 to −0.54)*	137772.44 (122777.49 to 154549.65)	52.6 (46.88 to 59.01)	101371.87 (86968.73 to 116777.92)	32.61 (27.98 to 37.57)	−1.68 (−1.77 to −1.6)*	
15–19 years	465454.24 (343219.7 to 623156.53)	182.15 (134.32 to 243.87)	466836.04 (339671.68 to 636547.55)	154.7 (112.56 to 210.95)	−0.53 (−0.56 to −0.5)*	236723.91 (213424.51 to 261263.12)	92.64 (83.52 to 102.24)	189724.63 (165436.37 to 214306.53)	62.87 (54.82 to 71.02)	−1.3 (−1.43 to −1.18)*	
20–24 years	641443.4 (474465.22 to 846839.92)	262.49 (194.16 to 346.54)	648386.27 (477583.95 to 868384.02)	219.22 (161.47 to 293.59)	−0.6 (−0.62 to −0.58)*	272980.73 (245607.53 to 302899.99)	111.71 (100.51 to 123.95)	234140.33 (204408.99 to 265921.52)	79.16 (69.11 to 89.91)	−1.23 (−1.34 to −1.14)*	
Sociodemographic index											
High-middle SDI	308390.64 (235463.35 to 397098.59)	207.75 (158.62 to 267.5)	200535.16 (150497.79 to 261425.15)	164.87 (123.73 to 214.93)	−0.78 (−0.8 to −0.76)*	118076.34 (110554.79 to 126852.5)	79.54 (74.47 to 85.45)	54900.46 (49869.87 to 60642.45)	45.14 (41 to 49.86)	−1.92 (−2.09 to −1.74)*	
High SDI	212524.08 (159076.74 to 275464.54)	237.82 (178.01 to 308.26)	171885.82 (128997.13 to 222493.15)	202.4 (151.9 to 262)	−0.58 (−0.62 to −0.55)*	52465.2 (49558.17 to 56254.14)	58.71 (55.46 to 62.95)	23361.29 (21076.19 to 26297.57)	27.51 (24.82 to 30.97)	−2.6 (−2.76 to −2.47)*	
Low-middle SDI	336762.86 (257051.79 to 429003.75)	193.33 (147.57 to 246.28)	408342.33 (307447.08 to 528367.05)	162.84 (122.6 to 210.7)	−0.58 (−0.62 to −0.55)*	195881.01 (166865.07 to 231514.99)	112.45 (95.79 to 132.91)	174999.62 (150382.85 to 200382.38)	69.79 (59.97 to 79.91)	−1.63 (−1.73 to −1.52)*	
Low SDI	113130.48 (86120.65 to 142176.04)	139.47 (106.17 to 175.27)	230948.76 (174870.9 to 292545.84)	125.2 (94.8 to 158.59)	−0.36 (−0.42 to −0.31)*	64978.27 (52472.01 to 78284.88)	80.1 (64.69 to 96.51)	123910.75 (95341.22 to 153359.25)	67.17 (51.68 to 83.14)	−0.58 (−0.64 to −0.52)*	
Middle SDI	440,502 (333019.35 to 563797.88)	164.2 (124.14 to 210.16)	402258.46 (299161.86 to 522393.34)	151.2 (112.45 to 196.36)	−0.27 (−0.3 to −0.24)*	215711.85 (196366.7 to 237011.11)	80.41 (73.2 to 88.35)	147602.17 (132416.65 to 165011.56)	55.48 (49.77 to 62.02)	−1.25 (−1.32 to −1.2)*	
Region											
Andean Latin America	5517.13 (4250.39 to 6975.24)	89.21 (68.73 to 112.79)	6831.84 (5129.84 to 8807.22)	83.7 (62.85 to 107.9)	−0.19 (−0.21 to −0.17)*	6543.6 (5316.08 to 7956.9)	105.81 (85.96 to 128.66)	7298.82 (5564.58 to 9297.15)	89.42 (68.17 to 113.9)	−0.6 (−0.69 to −0.5)*	
Australasia	9356.26 (6929.94 to 12230.95)	395.99 (293.3 to 517.65)	9759.52 (7262.1 to 12726.33)	367.77 (273.66 to 479.56)	−0.28 (−0.32 to −0.25)*	1495.48 (1314.81 to 1706.09)	63.29 (55.65 to 72.21)	982.59 (820.27 to 1171.95)	37.03 (30.91 to 44.16)	−2.11 (−2.53 to −1.74)*	
Caribbean	8127.3 (6242.51 to 10302.94)	151.7 (116.52 to 192.31)	8360.41 (6360.73 to 10750.79)	147.23 (112.01 to 189.32)	−0.06 (−0.09 to −0.03)*	6342.52 (4957.65 to 8500.91)	118.39 (92.54 to 158.67)	6454.59 (4069.37 to 9374.46)	113.67 (71.66 to 165.08)	−0.09 (−0.22 to −0.01)*	
Central Asia	16273.25 (12523.55 to 20878.09)	165.47 (127.34 to 212.29)	15434.81 (11684.49 to 19777.95)	140.23 (106.16 to 179.69)	−0.48 (−0.54 to −0.42)*	8822.19 (8142.94 to 9549.1)	89.7 (82.8 to 97.1)	5349.44 (4674.22 to 6069.69)	48.6 (42.47 to 55.14)	−2.1 (−2.28 to −1.91)*	
Central Europe	37301.06 (28397.8 to 47758.54)	265.4 (202.06 to 339.81)	23696.14 (17751.81 to 30474.82)	271.43 (203.34 to 349.07)	0.09 (0.02 to 0.14)*	7807.44 (7118.66 to 8658.15)	55.55 (50.65 to 61.6)	2400.81 (2004.51 to 2886.38)	27.5 (22.96 to 33.06)	−2.57 (−2.78 to −2.41)*	
Central Europe, Eastern Europe, and Central Asia	150949.04 (115284.41 to 194106.13)	320.06 (244.44 to 411.56)	83646.27 (63051.36 to 107479.78)	235.58 (177.58 to 302.7)	−1.01 (−1.07 to −0.95)*	60756.5 (57913.56 to 64087.49)	128.82 (122.79 to 135.88)	29996.32 (26707.93 to 34187.13)	84.48 (75.22 to 96.28)	−1.58 (−1.92 to −1.24)*	
Central Latin America	78330.51 (58458.9 to 103166.24)	286.17 (213.57 to 376.9)	78481.95 (58143.69 to 103942.98)	240.4 (178.1 to 318.39)	−0.63 (−0.7 to −0.55)*	38439.23 (36119.91 to 41219.19)	140.43 (131.96 to 150.59)	45445.31 (38896.41 to 52921.9)	139.21 (119.15 to 162.11)	−0.28 (−0.53 to −0.04)*	
Central Sub-Saharan Africa	6125.23 (4712.45 to 7728.79)	70.95 (54.59 to 89.53)	14151.03 (10901.44 to 17743.83)	65.8 (50.69 to 82.51)	−0.25 (−0.28 to −0.22)*	4763.96 (3263.28 to 6703.62)	55.18 (37.8 to 77.65)	10626.37 (7086.49 to 15214.37)	49.41 (32.95 to 70.75)	−0.24 (−0.35 to −0.11)*	
East Asia	216240.27 (162722.96 to 279209.26)	118.87 (89.45 to 153.48)	90107.67 (66108.23 to 121148.68)	81.2 (59.57 to 109.17)	−1.35 (−1.44 to −1.29)*	98535.29 (82793.94 to 118240.17)	54.17 (45.51 to 65)	22479.29 (18920.48 to 26347.83)	20.26 (17.05 to 23.74)	−3.53 (−3.72 to −3.21)*	
Eastern Europe	97374.73 (74111.95 to 125481.05)	418.38 (318.43 to 539.15)	44515.32 (33383.91 to 57942.12)	282.29 (211.7 to 367.43)	−1.31 (−1.35 to −1.26)*	44126.87 (42045.69 to 46437.15)	189.6 (180.66 to 199.52)	22246.07 (19407.01 to 25924.95)	141.07 (123.07 to 164.4)	−1.15 (−1.56 to −0.71)*	
Eastern Sub-Saharan Africa	31441.68 (23820.62 to 39840.21)	100.67 (76.27 to 127.56)	62868.98 (47462.3 to 79880.58)	89.84 (67.82 to 114.15)	−0.38 (−0.42 to −0.34)*	24189.4 (18124.2 to 31045.2)	77.45 (58.03 to 99.4)	47569.62 (35091.68 to 62559.26)	67.98 (50.15 to 89.4)	−0.45 (−0.5 to −0.41)*	
High-income	231933.25 (173646.6 to 301360.47)	232.97 (174.43 to 302.71)	183968.31 (138,211 to 238122.62)	198.61 (149.21 to 257.07)	−0.57 (−0.61 to −0.54)*	55934.62 (52606.42 to 60062.57)	56.19 (52.84 to 60.33)	25521.91 (23169.23 to 28714.84)	27.55 (25.01 to 31)	−2.48 (−2.63 to −2.35)*	
High-income Asia Pacific	43347.19 (31851.29 to 56522.3)	211.1 (155.11 to 275.26)	27700.73 (20359.3 to 36385.56)	212.52 (156.2 to 279.15)	0.04 (−0.07 to 0.13)	7534.6 (6742.21 to 8461.89)	36.69 (32.83 to 41.21)	3138.13 (2681.72 to 3726.34)	24.08 (20.57 to 28.59)	−1.5 (−1.75 to −1.33)*	
High-income North America	88575.09 (65089.5 to 117911.53)	297.23 (218.42 to 395.67)	72684.31 (53698.44 to 96345.56)	209.4 (154.7 to 277.57)	−1.31 (−1.44 to −1.2)*	28880.83 (27489.84 to 30593.38)	96.92 (92.25 to 102.66)	12008.01 (11042.35 to 13322.38)	34.59 (31.81 to 38.38)	−3.42 (−3.69 to −3.2)*	
Latin America and Caribbean	135837.93 (102151.22 to 176250.64)	215.94 (162.39 to 280.19)	118663.24 (88109.8 to 155197.05)	164.22 (121.94 to 214.78)	−0.94 (−1 to −0.88)*	81224.4 (77101.19 to 86,837)	129.12 (122.57 to 138.05)	78539.8 (69957.48 to 88978.56)	108.69 (96.82 to 123.14)	−0.68 (−0.86 to −0.48)*	
North Africa and Middle East	65365.52 (49266.24 to 85296.47)	121.28 (91.41 to 158.26)	100970.47 (75113.99 to 132760.32)	129.43 (96.28 to 170.18)	0.25 (0.22 to 0.28)*	20926.55 (17320.78 to 24781.63)	38.83 (32.14 to 45.98)	38973.46 (32488.59 to 47439.07)	49.96 (41.65 to 60.81)	0.93 (0.82 to 1.05)*	
Oceania	1018.24 (767.99 to 1366.3)	101.98 (76.91 to 136.84)	2271.02 (1720.12 to 3051.42)	122.09 (92.47 to 164.05)	0.63 (0.6 to 0.65)*	939.63 (700.19 to 1240.68)	94.1 (70.12 to 124.25)	2723.36 (1826.33 to 4004.34)	146.41 (98.18 to 215.27)	1.47 (1.28 to 1.62)*	
South Asia	382779.26 (290496.68 to 490708.73)	236.85 (179.75 to 303.64)	510412.67 (383560.99 to 660992.33)	200.52 (150.68 to 259.67)	−0.56 (−0.62 to −0.51)*	201156.32 (166597.61 to 243531.22)	124.47 (103.09 to 150.69)	177507.8 (145633.94 to 211421.16)	69.73 (57.21 to 83.06)	−2.03 (−2.19 to −1.89)*	
Southeast Asia	127354.95 (96561.62 to 165351.5)	171.93 (130.36 to 223.23)	133745.55 (99097.04 to 173267.33)	160.52 (118.94 to 207.95)	−0.22 (−0.3 to −0.16)*	42377.57 (36068.82 to 49237.72)	57.21 (48.69 to 66.47)	23792.88 (20624.49 to 27501.2)	28.56 (24.75 to 33.01)	−2.32 (−2.43 to −2.23)*	
Southeast Asia, East Asia, and Oceania	344613.47 (263313.22 to 443163.81)	134.1 (102.46 to 172.45)	226124.23 (167701.31 to 296622.9)	115.28 (85.5 to 151.22)	−0.53 (−0.56 to −0.49)*	141852.48 (123879.48 to 163572.03)	55.2 (48.2 to 63.65)	48995.53 (43260.66 to 55522.63)	24.98 (22.05 to 28.31)	−2.71 (−2.83 to −2.57)*	
Southern Latin America	15984.55 (12279.54 to 20459.27)	241.61 (185.61 to 309.24)	17047.98 (12859.08 to 22168.63)	226.98 (171.21 to 295.16)	−0.22 (−0.23 to −0.2)*	4155.3 (3693.92 to 4659.99)	62.81 (55.83 to 70.44)	3061.02 (2623.95 to 3545.59)	40.75 (34.94 to 47.21)	−1.56 (−1.87 to −1.25)*	
Southern Sub-Saharan Africa	23998.34 (18160.06 to 30881.84)	270.07 (204.37 to 347.54)	17864.01 (13410.23 to 23129.81)	168.47 (126.47 to 218.13)	−1.61 (−1.65 to −1.57)*	37628.86 (31789.03 to 43641.68)	423.47 (357.75 to 491.14)	23836.09 (15576.05 to 33645.2)	224.8 (146.9 to 317.3)	−2.11 (−2.27 to −1.95)*	
Sub-Saharan Africa	100460.96 (75812.91 to 128048.79)	126.05 (95.12 to 160.66)	190956.72 (143810.49 to 243032.52)	106.51 (80.21 to 135.56)	−0.56 (−0.62 to −0.5)*	85626.2 (73175.65 to 98452.15)	107.43 (91.81 to 123.53)	125702.01 (94,097 to 159819.88)	70.11 (52.49 to 89.14)	−1.62 (−1.78 to −1.5)*	
Tropical Latin America	43862.99 (32943.07 to 56582.93)	182.84 (137.32 to 235.86)	24989.03 (18529.45 to 33526.92)	96.97 (71.9 to 130.1)	−2.18 (−2.22 to −2.14)*	29899.05 (28121.21 to 31738.99)	124.63 (117.22 to 132.3)	19341.08 (18036.99 to 21011.27)	75.05 (69.99 to 81.53)	−1.65 (−1.93 to −1.38)*	
Western Europe	74670.17 (56362.41 to 96192.65)	185.56 (140.07 to 239.05)	56775.76 (43052.59 to 72457.17)	163.52 (124 to 208.69)	−0.38 (−0.43 to −0.34)*	13868.41 (12744.81 to 15304.72)	34.46 (31.67 to 38.03)	6332.16 (5570.81 to 7357.99)	18.24 (16.04 to 21.19)	−2.2 (−2.4 to −2)*	
Western Sub-Saharan Africa	38895.7 (29136.29 to 50002.01)	125.68 (94.15 to 161.57)	96072.69 (72346.24 to 123500.27)	124.45 (93.72 to 159.98)	−0.02 (−0.07 to 0.02)	19043.98 (15112.22 to 24376.58)	61.54 (48.83 to 78.77)	43669.94 (30200.07 to 60924.43)	56.57 (39.12 to 78.92)	−0.49 (−0.7 to −0.29)*	
*Physical violence by sharp object*											
Global	384613.13 (295479.43 to 501015.56)	50.49 (38.79 to 65.77)	345213.3 (264241.54 to 447914.6)	38 (29.09 to 49.31)	−0.97 (−0.99 to −0.96)*	307441.84 (286189.92 to 330298.97)	40.36 (37.57 to 43.36)	231468.38 (201349.44 to 267255.48)	25.48 (22.17 to 29.42)	−1.58 (−1.71 to −1.45)*	
Age group, years											
10–14 years	95689.19 (71291.88 to 129077.2)	36.53 (27.22 to 49.28)	83759.91 (62286.3 to 112202.12)	26.95 (20.04 to 36.09)	−1.04 (−1.06 to −1.03)*	44940.71 (40220.48 to 50121.51)	17.16 (15.36 to 19.14)	33469.43 (27764.68 to 40032.11)	10.77 (8.93 to 12.88)	−1.55 (−1.73 to −1.42)*	
15–19 years	127676.98 (96475.47 to 168215.01)	49.97 (37.76 to 65.83)	114329.56 (86,328 to 151002.93)	37.89 (28.61 to 50.04)	−0.94 (−0.96 to −0.93)*	107598.33 (99507.93 to 116006.63)	42.11 (38.94 to 45.4)	83577.25 (71250.28 to 99472.7)	27.7 (23.61 to 32.96)	−1.32 (−1.48 to −1.18)*	
20–24 years	161246.96 (121716.28 to 210826.37)	65.99 (49.81 to 86.27)	147123.83 (111086.15 to 190712.78)	49.74 (37.56 to 64.48)	−0.98 (−0.99 to −0.97)*	154902.8 (141921.87 to 169061.27)	63.39 (58.08 to 69.18)	114421.7 (100727.78 to 130824.56)	38.69 (34.06 to 44.23)	−1.72 (−1.93 to −1.56)*	
Sociodemographic index											
High-middle SDI	99312.51 (76093.61 to 130756.82)	66.9 (51.26 to 88.08)	57413.95 (43866.22 to 74571.64)	47.2 (36.06 to 61.31)	−1.2 (−1.21 to −1.18)*	62141.36 (57502.63 to 68327.68)	41.86 (38.74 to 46.03)	26219.99 (23999.32 to 28790.84)	21.56 (19.73 to 23.67)	−2.05 (−2.34 to −1.75)*	
High SDI	44046.59 (33563.36 to 57138.55)	49.29 (37.56 to 63.94)	32830.85 (24982.59 to 42637.71)	38.66 (29.42 to 50.21)	−0.87 (−0.91 to −0.82)*	28470.98 (27432.97 to 29619.16)	31.86 (30.7 to 33.15)	11000.63 (10428.36 to 11634.23)	12.95 (12.28 to 13.7)	−3.05 (−3.26 to −2.84)*	
Low-middle SDI	71128.11 (54713.42 to 92167.46)	40.83 (31.41 to 52.91)	83932.79 (64347.26 to 108791.31)	33.47 (25.66 to 43.38)	−0.66 (−0.68 to −0.64)*	59864.5 (52263.13 to 68268.83)	34.37 (30 to 39.19)	62526.98 (54116.49 to 72302.89)	24.93 (21.58 to 28.83)	−1.14 (−1.34 to −0.99)*	
Low SDI	28292.98 (21751.51 to 36512.04)	34.88 (26.81 to 45.01)	58031.33 (44536.59 to 75417.32)	31.46 (24.14 to 40.88)	−0.35 (−0.37 to −0.33)*	25046.04 (20213.86 to 30427.03)	30.88 (24.92 to 37.51)	57873.56 (39544.14 to 80885.73)	31.37 (21.44 to 43.85)	0.04 (−1.35 to 0.79)	
Middle SDI	141541.91 (108314.83 to 185620.94)	52.76 (40.38 to 69.19)	112690.51 (85730.92 to 146640.11)	42.36 (32.22 to 55.12)	−0.75 (−0.79 to −0.72)*	131586.72 (119886.15 to 145463.19)	49.05 (44.69 to 54.22)	73519.81 (65278.57 to 83241.81)	27.63 (24.54 to 31.29)	−1.9 (−2.09 to −1.79)*	
Region											
Andean Latin America	3136.33 (2386.98 to 4075.27)	50.71 (38.6 to 65.9)	3843.31 (2930.95 to 4972.63)	47.09 (35.91 to 60.92)	−0.23 (−0.25 to −0.22)*	1744.28 (1283.8 to 2270.83)	28.2 (20.76 to 36.72)	2630.69 (1903.81 to 3527.25)	32.23 (23.32 to 43.21)	0.42 (0.28 to 0.54)*	
Australasia	1357.93 (1018.54 to 1748.02)	57.47 (43.11 to 73.98)	1314.66 (993.01 to 1693.61)	49.54 (37.42 to 63.82)	−0.52 (−0.54 to −0.5)*	905.25 (812.16 to 1014.32)	38.31 (34.37 to 42.93)	482.89 (414.09 to 562.32)	18.2 (15.6 to 21.19)	−2.86 (−3.71 to −2.01)*	
Caribbean	5423.45 (4156.91 to 7297.9)	101.23 (77.59 to 136.22)	5087.64 (3884.35 to 6778.15)	89.59 (68.4 to 119.36)	−0.4 (−0.44 to −0.38)*	7398.14 (6389.75 to 8679.22)	138.09 (119.27 to 162)	6611.65 (4646.93 to 9298.26)	116.43 (81.83 to 163.74)	−0.65 (−0.85 to −0.51)*	
Central Asia	7287.28 (5611.01 to 9591.53)	74.1 (57.05 to 97.53)	7059.27 (5388.84 to 9280.19)	64.14 (48.96 to 84.31)	−0.47 (−0.54 to −0.41)*	4294.03 (3925.9 to 4647.43)	43.66 (39.92 to 47.26)	2946.6 (2501.71 to 3469.34)	26.77 (22.73 to 31.52)	−1.48 (−1.86 to −1.04)*	
Central Europe	12009.96 (9181.55 to 16171.95)	85.45 (65.33 to 115.07)	7082.97 (5400.74 to 9539.75)	81.13 (61.86 to 109.27)	−0.17 (−0.26 to −0.09)*	3978.55 (3710.55 to 4262.58)	28.31 (26.4 to 30.33)	1064.9 (912.08 to 1237.82)	12.2 (10.45 to 14.18)	−3.11 (−3.43 to −2.83)*	
Central Europe, Eastern Europe, and Central Asia	58503.42 (44468.02 to 79092.85)	124.04 (94.29 to 167.7)	31672.03 (24162.51 to 42231.39)	89.2 (68.05 to 118.94)	−1.08 (−1.14 to −1.02)*	30981.54 (29561.93 to 32392.55)	65.69 (62.68 to 68.68)	12286.88 (10784.73 to 14028.44)	34.6 (30.37 to 39.51)	−2.12 (−2.76 to −1.63)*	
Central Latin America	43752.56 (32639.62 to 60114.72)	159.84 (119.24 to 219.62)	40959.19 (30295.44 to 55222.74)	125.46 (92.8 to 169.16)	−0.85 (−0.91 to −0.8)*	17202.12 (15969.72 to 18574.12)	62.84 (58.34 to 67.86)	25506.88 (21240.33 to 30621.87)	78.13 (65.06 to 93.8)	0.51 (0.07 to 0.79)*	
Central Sub-Saharan Africa	2207.92 (1712.22 to 2835.46)	25.58 (19.83 to 32.84)	5222.25 (4052.53 to 6659.68)	24.28 (18.84 to 30.97)	−0.17 (−0.2 to −0.14)*	1923.37 (1238.07 to 2926.55)	22.28 (14.34 to 33.9)	4773.67 (2787.82 to 7943.12)	22.2 (12.96 to 36.94)	3.84 (2.16 to 6.69)*	
East Asia	61335.23 (46749.48 to 79987.74)	33.72 (25.7 to 43.97)	24696.28 (18593.54 to 32630.83)	22.25 (16.76 to 29.4)	−1.46 (−1.55 to −1.38)*	76625.72 (62421.7 to 95412.64)	42.12 (34.31 to 52.45)	15223.6 (12291.94 to 18270.61)	13.72 (11.08 to 16.46)	−3.83 (−4 to −3.61)*	
Eastern Europe	39206.18 (29637.56 to 53177.69)	168.45 (127.34 to 228.49)	17529.78 (13129.69 to 23423.41)	111.16 (83.26 to 148.54)	−1.38 (−1.44 to −1.33)*	22708.96 (21,516 to 23813.19)	97.57 (92.45 to 102.32)	8275.38 (7010.41 to 9665.62)	52.48 (44.46 to 61.29)	−2.07 (−3.1 to −1.38)*	
Eastern Sub-Saharan Africa	12408.68 (9444.77 to 16079.23)	39.73 (30.24 to 51.48)	24541.58 (18765.76 to 31661.19)	35.07 (26.82 to 45.24)	−0.42 (−0.45 to −0.39)*	10191.59 (7101.18 to 14128.04)	32.63 (22.74 to 45.23)	22537.12 (16164.67 to 29702.6)	32.21 (23.1 to 42.45)	0.48 (−0.37 to 2.02)	
High-income	48861.96 (37311.6 to 63310.93)	49.08 (37.48 to 63.6)	36411.54 (27717.76 to 47306.2)	39.31 (29.92 to 51.07)	−0.79 (−0.83 to −0.75)*	28228.04 (27109.56 to 29412.33)	28.35 (27.23 to 29.54)	13309.45 (12561.65 to 14098.07)	14.37 (13.56 to 15.22)	−2.44 (−2.71 to −2.2)*	
High-income Asia Pacific	7110.95 (5372.6 to 9247.74)	34.63 (26.16 to 45.04)	4428.6 (3319.14 to 5803.71)	33.98 (25.46 to 44.53)	−0.05 (−0.12 to 0.01)	2847.5 (2405.98 to 3370.19)	13.87 (11.72 to 16.41)	732.7 (593.13 to 929.12)	5.62 (4.55 to 7.13)	−3.06 (−3.41 to −2.74)*	
High-income North America	19508.49 (14395.71 to 26046.25)	65.46 (48.31 to 87.4)	13944.32 (10413.95 to 18223.74)	40.17 (30 to 52.5)	−1.83 (−2.05 to −1.65)*	17539.64 (16938.72 to 18185.49)	58.86 (56.84 to 61.02)	6714.08 (6397.55 to 7082.75)	19.34 (18.43 to 20.41)	−3.83 (−4.16 to −3.53)*	
Latin America and Caribbean	70403.47 (53014.24 to 95021.15)	111.92 (84.28 to 151.06)	58898.1 (44207.18 to 78334.39)	81.51 (61.18 to 108.41)	−1.11 (−1.18 to −1.06)*	43634.26 (41447.39 to 45982.69)	69.37 (65.89 to 73.1)	55439.2 (49481.12 to 62723.51)	76.72 (68.48 to 86.81)	0.29 (−0.31 to 0.69)	
North Africa and Middle East	21524.11 (16455.8 to 27897.86)	39.94 (30.53 to 51.76)	29471.48 (22439.52 to 38384.78)	37.78 (28.76 to 49.2)	−0.17 (−0.19 to −0.16)*	5953.39 (5036.02 to 6927.2)	11.05 (9.34 to 12.85)	7410.56 (6068.95 to 9055.9)	9.5 (7.78 to 11.61)	−0.28 (−1.07 to 0.46)	
Oceania	369.97 (285.69 to 476.11)	37.05 (28.61 to 47.68)	764.87 (591.23 to 988.72)	41.12 (31.78 to 53.15)	0.37 (0.33 to 0.4)*	322.57 (201.58 to 498.05)	32.31 (20.19 to 49.88)	1058.66 (597.3 to 1715.27)	56.91 (32.11 to 92.21)	2.02 (1.64 to 2.35)*	
South Asia	47737.86 (36720.63 to 61214.99)	29.54 (22.72 to 37.88)	63428.91 (48615.31 to 82690.65)	24.92 (19.1 to 32.49)	−0.56 (−0.64 to −0.5)*	30438.73 (24,679 to 36819.12)	18.83 (15.27 to 22.78)	29372.32 (23866.42 to 35319.68)	11.54 (9.38 to 13.88)	−1.8 (−1.93 to −1.64)*	
Southeast Asia	39865.5 (30364.65 to 51532.83)	53.82 (40.99 to 69.57)	34644.12 (26438.36 to 44724.77)	41.58 (31.73 to 53.68)	−0.89 (−0.94 to −0.85)*	38316.99 (32712.02 to 44636.4)	51.73 (44.16 to 60.26)	18632.36 (15525.08 to 21714.78)	22.36 (18.63 to 26.06)	−2.84 (−2.95 to −2.74)*	
Southeast Asia, East Asia, and Oceania	101570.71 (78179.04 to 131921.86)	39.52 (30.42 to 51.33)	60105.27 (45746.82 to 78217.6)	30.64 (23.32 to 39.88)	−0.89 (−0.93 to −0.86)*	115265.28 (99921.51 to 134956.68)	44.85 (38.88 to 52.52)	34914.62 (30277.99 to 39670.3)	17.8 (15.44 to 20.22)	−3.13 (−3.24 to −3)*	
Southern Latin America	6,188 (4746.51 to 8316.26)	93.53 (71.74 to 125.7)	5752.71 (4372.34 to 7447.08)	76.59 (58.21 to 99.15)	−0.68 (−0.7 to −0.67)*	1444.02 (1257.01 to 1643.95)	21.83 (19 to 24.85)	2235.88 (1915.58 to 2590.99)	29.77 (25.5 to 34.5)	1.2 (0.55 to 1.7)*	
Southern Sub-Saharan Africa	9138.72 (6799.84 to 12311.81)	102.85 (76.52 to 138.55)	7970.96 (5965.29 to 10725.44)	75.17 (56.26 to 101.15)	−1.03 (−1.09 to −0.97)*	25937.3 (21683.33 to 30758.89)	291.89 (244.02 to 346.16)	14026.8 (8136.48 to 20866.15)	132.28 (76.73 to 196.79)	−2.92 (−3.17 to −2.75)*	
Sub-Saharan Africa	36011.61 (27399.92 to 47027.75)	45.18 (34.38 to 59.01)	65225.97 (49717.56 to 84451.05)	36.38 (27.73 to 47.1)	−0.73 (−0.77 to −0.71)*	52940.61 (45178.96 to 61119.31)	66.42 (56.69 to 76.69)	78735.35 (54623.01 to 108820.09)	43.92 (30.47 to 60.7)	−1.24 (−1.58 to −0.94)*	
Tropical Latin America	18091.12 (13614.74 to 24072.46)	75.41 (56.75 to 100.34)	9007.96 (6790.67 to 11745.34)	34.96 (26.35 to 45.58)	−2.65 (−2.75 to −2.56)*	17289.72 (16356.09 to 18366.53)	72.07 (68.18 to 76.56)	20689.97 (19266.61 to 22243.8)	80.29 (74.76 to 86.32)	0.31 (−0.13 to 0.97)	
Western Europe	14696.59 (11264.77 to 18989.18)	36.52 (27.99 to 47.19)	10971.25 (8456.98 to 14218.46)	31.6 (24.36 to 40.95)	−0.49 (−0.52 to −0.47)*	5491.64 (5143.27 to 5847.75)	13.65 (12.78 to 14.53)	3143.91 (2918.83 to 3399.72)	9.06 (8.41 to 9.79)	−1.94 (−2.49 to −1.33)*	
Western Sub-Saharan Africa	12256.28 (9293.4 to 16047.04)	39.6 (30.03 to 51.85)	27491.19 (20744.01 to 35854.22)	35.61 (26.87 to 46.45)	−0.37 (−0.39 to −0.35)*	14888.35 (11241.06 to 20262.66)	48.11 (36.32 to 65.47)	37397.76 (22355.69 to 59160.11)	48.45 (28.96 to 76.64)	0.07 (−0.29 to 0.42)	
*Sexual violence*											
Global	36550027.37 (27495977.89 to 46652637.31)	4797.76 (3609.28 to 6123.89)	37949685.16 (28911179.92 to 47983875.13)	4177.7 (3182.69 to 5282.31)	−0.47 (−0.48 to −0.46)*	377435.45 (236686.1 to 548871.92)	49.54 (31.07 to 72.05)	389078.01 (247342.16 to 566391.98)	42.83 (27.23 to 62.35)	−0.49 (−0.51 to −0.48)*	
Age group, years											
10–14 years	5462351.79 (3809372.33 to 7399104.85)	2085.51 (1454.41 to 2824.96)	5960308.35 (4157242.54 to 8055325.24)	1917.41 (1337.37 to 2591.37)	−0.27 (−0.28 to −0.26)*	57126.65 (33173.47 to 87127.2)	21.81 (12.67 to 33.26)	62221.71 (36033.79 to 94905.96)	20.02 (11.59 to 30.53)	−0.28 (−0.3 to −0.26)*	
15–19 years	13002971.62 (8769493.23 to 17942830.2)	5088.66 (3431.9 to 7021.85)	13045205.62 (8847255.86 to 17943891.84)	4323.06 (2931.9 to 5946.43)	−0.54 (−0.56 to −0.52)*	134821.21 (77213.33 to 212392.01)	52.76 (30.22 to 83.12)	134406.27 (77580.37 to 210932.58)	44.54 (25.71 to 69.9)	−0.56 (−0.58 to −0.54)*	
20–24 years	18084703.96 (12779285.9 to 24444873.03)	7400.63 (5229.55 to 10003.35)	18944171.2 (13505361.53 to 25480214.94)	6404.9 (4566.07 to 8614.69)	−0.5 (−0.51 to −0.49)*	185487.59 (109757.22 to 278221.7)	75.91 (44.91 to 113.85)	192450.03 (114218.88 to 286305.99)	65.07 (38.62 to 96.8)	−0.53 (−0.54 to −0.52)*	
Sociodemographic index											
High-middle SDI	7857473.35 (5917615.75 to 10054130.82)	5293.17 (3986.39 to 6772.94)	5921992.24 (4469160.75 to 7568220.9)	4868.81 (3674.36 to 6222.27)	−0.29 (−0.31 to −0.28)*	81613.59 (51160.25 to 119567.88)	54.98 (34.46 to 80.55)	61225.73 (38307.27 to 89569.97)	50.34 (31.49 to 73.64)	−0.31 (−0.33 to −0.3)*	
High SDI	3412789.96 (2532457.4 to 4449342.44)	3819.07 (2833.93 to 4979.02)	3251185.45 (2423978.22 to 4221627.96)	3828.45 (2854.37 to 4971.19)	0.01 (−0.01 to 0.02)	34605.23 (21564.95 to 50526.87)	38.72 (24.13 to 56.54)	32964.14 (20449.36 to 47626.74)	38.82 (24.08 to 56.08)	0.01 (−0.01 to 0.02)	
Low-middle SDI	6904523.03 (5163247.89 to 8816524.9)	3963.68 (2964.07 to 5061.31)	9052638.72 (6862303.87 to 11434734.22)	3609.96 (2736.51 to 4559.87)	−0.33 (−0.36 to −0.3)*	70669.19 (44376.49 to 102948.35)	40.57 (25.48 to 59.1)	92081.9 (58402.21 to 133210.51)	36.72 (23.29 to 53.12)	−0.36 (−0.38 to −0.33)*	
Low SDI	3636210.34 (2729382.7 to 4592383.5)	4482.67 (3364.74 to 5661.43)	7998727.05 (6,162,230 to 9979921.71)	4336.15 (3340.57 to 5410.16)	−0.12 (−0.14 to −0.11)*	37357.01 (23490.57 to 53825.74)	46.05 (28.96 to 66.36)	82097.91 (52243.89 to 118046.23)	44.51 (28.32 to 63.99)	−0.12 (−0.14 to −0.11)*	
Middle SDI	14719860.74 (11096443.88 to 18710133.47)	5486.99 (4136.32 to 6974.41)	11697710.56 (8828965.61 to 14877967.21)	4396.92 (3318.62 to 5592.32)	−0.75 (−0.77 to −0.74)*	152993.57 (94820.63 to 224208.37)	57.03 (35.35 to 83.58)	120425.74 (75689.66 to 175755.75)	45.27 (28.45 to 66.06)	−0.78 (−0.8 to −0.77)*	
Region											
Andean Latin America	170472.11 (125483.55 to 217783.29)	2756.48 (2029.03 to 3521.49)	241936.75 (186766.1 to 305567.95)	2964.05 (2288.13 to 3743.61)	0.25 (0.24 to 0.26)*	1713.94 (1052.88 to 2477.25)	27.71 (17.02 to 40.06)	2430.76 (1535.16 to 3522.67)	29.78 (18.81 to 43.16)	0.25 (0.24 to 0.26)*	
Australasia	75473.84 (54273.91 to 99205.93)	3194.3 (2297.05 to 4198.72)	83604.93 (60533.26 to 109185.32)	3150.46 (2281.06 to 4114.4)	−0.05 (−0.05 to −0.04)*	758.49 (464.46 to 1125.72)	32.1 (19.66 to 47.64)	839.88 (513.53 to 1241.63)	31.65 (19.35 to 46.79)	−0.05 (−0.06 to −0.05)*	
Caribbean	189932.52 (140387.94 to 243035.49)	3545.2 (2620.42 to 4536.4)	226883.62 (168028.51 to 290022.49)	3995.42 (2958.98 to 5107.29)	0.43 (0.41 to 0.45)*	1920.89 (1183.92 to 2800.89)	35.85 (22.1 to 52.28)	2304.15 (1419.77 to 3383.17)	40.58 (25 to 59.58)	0.44 (0.42 to 0.47)*	
Central Asia	143958.98 (105892.64 to 185263.08)	1463.78 (1076.72 to 1883.76)	168541.78 (124179.13 to 216962.32)	1531.24 (1128.2 to 1971.16)	0.15 (0.14 to 0.16)*	1429.44 (886.12 to 2080.96)	14.53 (9.01 to 21.16)	1671.35 (1039.83 to 2441.97)	15.18 (9.45 to 22.19)	0.14 (0.13 to 0.15)*	
Central Europe	277375.35 (202684.83 to 358823.31)	1973.58 (1442.14 to 2553.1)	176423.75 (128936.67 to 230362.35)	2020.83 (1476.89 to 2638.66)	0.08 (0.07 to 0.09)*	2762.29 (1721.39 to 4002.65)	19.65 (12.25 to 28.48)	1753.48 (1096.35 to 2548.04)	20.09 (12.56 to 29.19)	0.07 (0.07 to 0.08)*	
Central Europe, Eastern Europe, and Central Asia	879562.68 (640916.12 to 1143636.15)	1864.94 (1358.93 to 2424.85)	654957.75 (479572.78 to 851167.51)	1844.61 (1350.66 to 2397.21)	−0.05 (−0.06 to −0.04)*	8751.43 (5447.46 to 12796.48)	18.56 (11.55 to 27.13)	6508.91 (4064.48 to 9486.49)	18.33 (11.45 to 26.72)	−0.05 (−0.06 to −0.04)*	
Central Latin America	707412.24 (525571.59 to 917939.73)	2584.41 (1920.08 to 3353.53)	867324.28 (644346.6 to 1122584.21)	2656.74 (1973.73 to 3438.64)	0.09 (0.08 to 0.1)*	7088.97 (4384.68 to 10375.34)	25.9 (16.02 to 37.9)	8675.32 (5391.51 to 12719.49)	26.57 (16.51 to 38.96)	0.09 (0.08 to 0.1)*	
Central Sub-Saharan Africa	628653.27 (477461.57 to 790909.28)	7282.01 (5530.68 to 9161.5)	1554491.63 (1182493.66 to 1953706.83)	7228.5 (5498.68 to 9084.87)	0 (−0.02 to 0.02)	6586.65 (4132.19 to 9574.95)	76.3 (47.87 to 110.91)	16280.13 (10240.56 to 23649.54)	75.7 (47.62 to 109.97)	−0.01 (−0.03 to 0.01)	
East Asia	16782522.97 (12692981.04 to 21361063.9)	9225.46 (6977.41 to 11742.31)	10134507.07 (7676970.35 to 12868292.8)	9132.49 (6917.93 to 11595.98)	−0.05 (−0.07 to −0.03)*	177107.41 (110467.32 to 261971.9)	97.36 (60.72 to 144.01)	106892.99 (66682.55 to 158059.92)	96.32 (60.09 to 142.43)	−0.05 (−0.07 to −0.03)*	
Eastern Europe	458228.35 (332419.09 to 601058.14)	1968.84 (1428.28 to 2582.53)	309992.23 (224847.45 to 405225.95)	1965.77 (1425.84 to 2569.68)	0 (−0.02 to 0.01)	4559.7 (2795.42 to 6687.46)	19.59 (12.01 to 28.73)	3084.07 (1888.38 to 4526.3)	19.56 (11.97 to 28.7)	0 (−0.02 to 0.01)	
Eastern Sub-Saharan Africa	1761226.33 (1329428.8 to 2231635.47)	5638.87 (4256.4 to 7144.97)	3709165.08 (2899629.95 to 4552839.91)	5300.4 (4143.57 to 6506.01)	−0.21 (−0.23 to −0.19)*	18210.31 (11463.29 to 26421.19)	58.3 (36.7 to 84.59)	38273.19 (24763.72 to 55355.63)	54.69 (35.39 to 79.1)	−0.22 (−0.24 to −0.2)*	
High-income	3568333.92 (2627948.21 to 4691873.09)	3584.36 (2639.75 to 4712.95)	3300176.29 (2427096.84 to 4323477.1)	3562.79 (2620.23 to 4667.52)	−0.02 (−0.03 to −0.01)*	35968.67 (22292.19 to 52606.74)	36.13 (22.39 to 52.84)	33288.79 (20663.35 to 48620.23)	35.94 (22.31 to 52.49)	−0.02 (−0.03 to −0.01)*	
High-income Asia Pacific	620424.66 (450628.83 to 816698.49)	3021.41 (2194.52 to 3977.25)	399906.73 (290177.12 to 522020.38)	3068.11 (2226.26 to 4004.97)	0.05 (0.04 to 0.06)*	6225.43 (3797.79 to 9141.7)	30.32 (18.49 to 44.52)	4007.28 (2436.9 to 5876.58)	30.74 (18.7 to 45.09)	0.04 (0.04 to 0.05)*	
High-income North America	993226.68 (724754.39 to 1324508.89)	3332.96 (2432.05 to 4444.64)	1170076.85 (859085.27 to 1530297.41)	3370.98 (2475.02 to 4408.78)	0.04 (0 to 0.08)*	9997.34 (6132.22 to 14722.13)	33.55 (20.58 to 49.4)	11796.71 (7263.66 to 17377.55)	33.99 (20.93 to 50.06)	0.05 (0.01 to 0.09)*	
Latin America and Caribbean	1775928.55 (1321003.4 to 2285949.99)	2823.22 (2100.02 to 3,634)	2135552.12 (1592475.71 to 2749873.05)	2955.48 (2203.9 to 3805.67)	0.16 (0.16 to 0.17)*	17838.49 (11081.59 to 25967.58)	28.36 (17.62 to 41.28)	21426.12 (13455.11 to 31069.31)	29.65 (18.62 to 43)	0.16 (0.15 to 0.16)*	
North Africa and Middle East	2028691.31 (1512567.77 to 2590722.22)	3763.97 (2806.37 to 4806.75)	3044719.37 (2306145.13 to 3858621.82)	3902.85 (2956.12 to 4946.15)	0.13 (0.12 to 0.13)*	20558.06 (12862.99 to 29495.03)	38.14 (23.87 to 54.72)	30817.46 (19206.4 to 44013.25)	39.5 (24.62 to 56.42)	0.12 (0.12 to 0.13)*	
Oceania	88776.27 (67863.02 to 112473.44)	8,891 (6796.52 to 11264.28)	168615.9 (128584.12 to 213549.07)	9064.82 (6912.71 to 11480.44)	0.07 (0.06 to 0.07)*	940.79 (586.69 to 1392.02)	94.22 (58.76 to 139.41)	1786.69 (1109.33 to 2654.22)	96.05 (59.64 to 142.69)	0.07 (0.06 to 0.07)*	
South Asia	4968444.07 (3629339.65 to 6438262.19)	3074.35 (2245.74 to 3983.84)	7240740.88 (5438329.45 to 9194114.5)	2844.56 (2136.47 to 3611.95)	−0.28 (−0.31 to −0.24)*	50125.03 (30870.95 to 73199.7)	31.02 (19.1 to 45.29)	72805.53 (45137.62 to 104328.95)	28.6 (17.73 to 40.99)	−0.29 (−0.32 to −0.25)*	
Southeast Asia	2876427.32 (2140327.61 to 3681450.73)	3883.28 (2889.52 to 4970.09)	3386636.11 (2523984.88 to 4325082.46)	4064.61 (3029.26 to 5190.92)	0.19 (0.16 to 0.23)*	29305.69 (17923.76 to 42906.86)	39.56 (24.2 to 57.93)	34523.07 (21314.02 to 50317.12)	41.43 (25.58 to 60.39)	0.2 (0.16 to 0.24)*	
Southeast Asia, East Asia, and Oceania	19747726.55 (14903063.41 to 25038573.57)	7684.36 (5799.17 to 9743.17)	13689759.08 (10357981.68 to 17,309,312)	6979.15 (5280.58 to 8824.43)	−0.34 (−0.36 to −0.32)*	207353.9 (129095.41 to 305416.45)	80.69 (50.23 to 118.85)	143202.76 (89309.28 to 210412.48)	73.01 (45.53 to 107.27)	−0.35 (−0.37 to −0.33)*	
Southern Latin America	206937.05 (150733.34 to 272020.19)	3127.88 (2278.35 to 4111.61)	242987.89 (174882.7 to 320584.11)	3235.19 (2328.42 to 4268.32)	0.12 (0.12 to 0.12)*	2084.26 (1261.99 to 3119.8)	31.5 (19.08 to 47.16)	2443.06 (1483.45 to 3646.86)	32.53 (19.75 to 48.55)	0.11 (0.11 to 0.12)*	
Southern Sub-Saharan Africa	288381.59 (215861.18 to 368152.44)	3245.4 (2429.26 to 4143.12)	354363.83 (275652.27 to 442012.38)	3341.96 (2599.64 to 4168.56)	0.1 (0.06 to 0.13)*	2919.03 (1786.32 to 4240.56)	32.85 (20.1 to 47.72)	3583.73 (2277.67 to 5137.57)	33.8 (21.48 to 48.45)	0.09 (0.06 to 0.13)*	
Sub-Saharan Africa	3581340.29 (2685304.19 to 4539576.91)	4493.49 (3369.24 to 5695.79)	7883779.66 (6039606.97 to 9849998.53)	4397.39 (3368.76 to 5494.11)	−0.07 (−0.08 to −0.06)*	36839.87 (22889.78 to 53445.69)	46.22 (28.72 to 67.06)	81028.45 (51317.57 to 117031.37)	45.2 (28.62 to 65.28)	−0.07 (−0.09 to −0.06)*	
Tropical Latin America	708111.69 (523482.2 to 931869.21)	2951.66 (2182.06 to 3884.37)	799407.47 (591148.62 to 1067335.13)	3102.07 (2293.93 to 4141.75)	0.17 (0.16 to 0.18)*	7114.69 (4397.64 to 10546.35)	29.66 (18.33 to 43.96)	8015.88 (4962.44 to 11872.19)	31.11 (19.26 to 46.07)	0.17 (0.16 to 0.17)*	
Western Europe	1672271.7 (1221422.13 to 2228871.92)	4155.76 (3035.36 to 5538.97)	1403599.89 (1030173.7 to 1867740.67)	4042.63 (2967.1 to 5379.45)	−0.09 (−0.1 to −0.09)*	16903.15 (10427.91 to 25272.82)	42.01 (25.91 to 62.81)	14201.86 (8735.58 to 21198.33)	40.9 (25.16 to 61.06)	−0.09 (−0.09 to −0.08)*	
Western Sub-Saharan Africa	903079.1 (666058.69 to 1167999.76)	2918.05 (2152.18 to 3774.06)	2265759.12 (1675976.46 to 2922839.82)	2935.09 (2171.08 to 3786.28)	0.01 (0 to 0.03)	9123.87 (5640.96 to 13254.71)	29.48 (18.23 to 42.83)	22891.39 (14168.47 to 33267.07)	29.65 (18.35 to 43.09)	0.02 (0 to 0.03)*	

*Indicates that the data is significantly different from zero at alpha = 0.05 level. AAPC, average annual percentage change. DALYs, disability-adjusted life-years.

PVSO prevalence also decreased between 1990 and 2019 in all groups (Table 2), and the decrease was greater than for PVF, PVOM and SV, with the greatest decrease in 10–14-year-olds [from 36.53, (27.22–49.28) in 1990 to 26.95, (20.04–36.09) in 2019; AAPC -1.04, (−1.06 to −1.03)].

PVF, PVOM, PVSO and SV DALYs declined between 1990 and 2019 for all three age groups (Table 2). The greatest decline in DALYs was in 20-24-year-olds for PVSO [from 63.39, (58.08–69.18) in 1990 to 38.69, (34.06–44.23) in 2019; AAPC -1.72, (−1.93 to −1.56)]. Table 2 shows PVF, PVOM and SV DALYs for 10–14-year-olds, 15-19-year-olds and 20-24-year-olds in 1990 and 2019.

### Global trends by SDI

By SDI quintile, SV prevalence increased in high SDI countries (AAPC 0.01, −0.01 to 0.02) (Table 2) and PVF prevalence increased in high SDI (AAPC 0.14, 0.09 to 0.18) (Table 2), low-middle SDI (AAPC 1.13, 1.08 to 1.17) and low SDI (AAPC 1.45, 1.4 to 1.49) countries between 1990 and 2019. PVF, PVOM, PVSO and SV prevalence decreased in the remaining quintiles (high-middle SDI, low SDI, middle SDI) over the same period (Table 2).

### Regional trends

Regionally, PVF prevalence decreased most in East Asia (AAPC -0.9505, −1.0011 to −0.8975) between 1990 and 2019, and increased most in Andean Latin America (AAPC 2.6155, 2.5711 to 2.6611) (Table 2). PVOM prevalence decreased most in Tropical Latin America (AAPC -2.1753, −2.2161 to −2.1424) and increased most in Oceania (AAPC 0.6275, 0.6036 to 0.6498) (Table 2). PVSO prevalence decreased most in Tropical Latin America (AAPC -2.6486, −2.7468 to −2.5627) and increased most in Oceania (AAPC 0.3726, 0.333 to 0.4046) (Table 2). SV prevalence decreased most in South Asia (AAPC -0.277, −0.3089 to −0.244) and increased most in the Caribbean (AAPC 0.4267, 0.4069 to 0.4495) (Table 2).

Between 1990 and 2019, PVF DALYs decreased most in Southern Sub-Saharan Africa (AAPC -5.1803, −5.3584 to −5.0267) and increased most in Oceania (AAPC 2.7733, 2.1927 to 3.3844). PVOM prevalence decreased most in East Asia (AAPC -3.5295, −3.7168 to −3.2075) and increased most in Oceania (AAPC 1.4673, 1.2832 to 1.618). PVSO prevalence decreased most in East Asia (AAPC -3.8335, −3.9965 to −3.6118) and increased most in Central Sub-Saharan Africa (AAPC 3.8443, 2.1632 to 6.6868). SV prevalence decreased most in South Asia (AAPC -0.2855, −0.3171 to −0.2546) and increased most in the Caribbean (AAPC 0.4416, 0.4211 to 0.4654). Table 2 shows regional PVF, PVOM, PVSO and SV prevalence, DALYs and AAPCs from 1990 to 2019. Table 2 shows PVF, PVOM, PVSO and SV AAPCs for 21 regions.

### National trends

**Figure 2:**
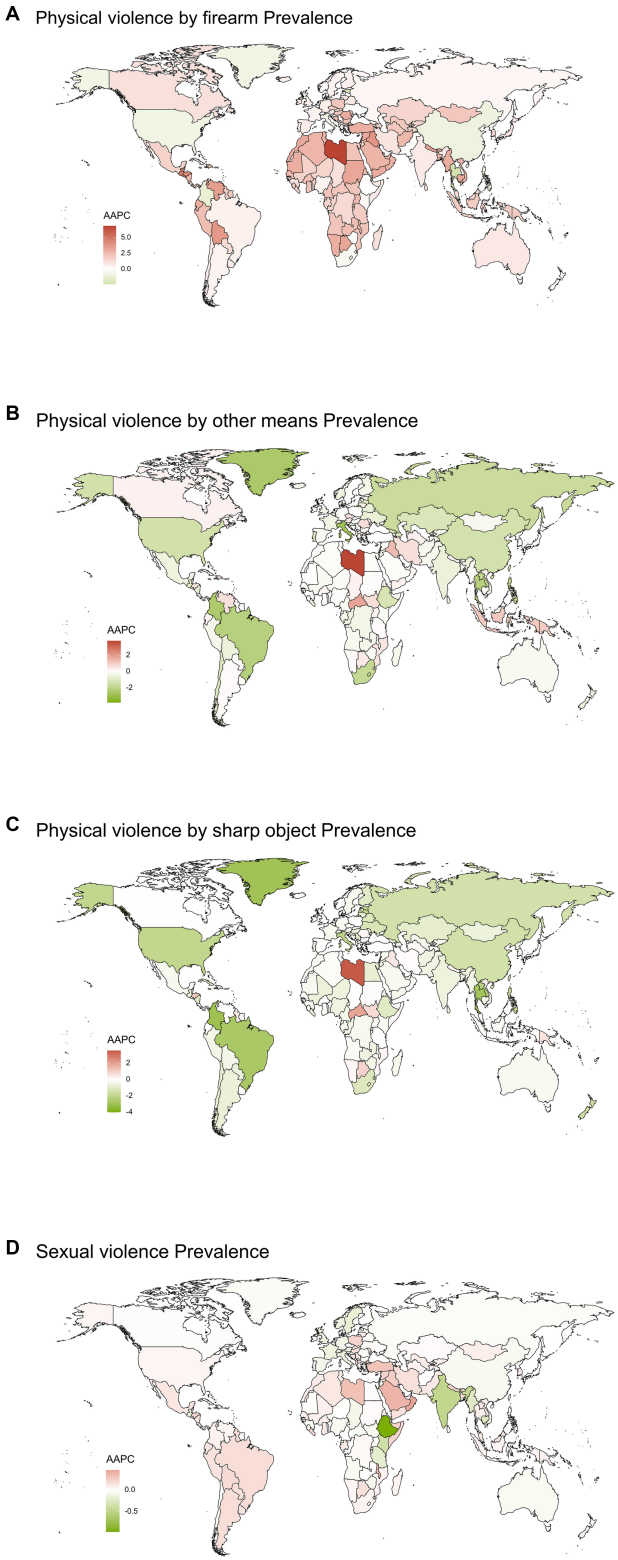
Global map of average annual percentage changes in prevalence of Physical violence by firearm **(A)**, Physical violence by other means **(B)**, Physical violence by sharp object **(C)** and Sexual violence **(D)** from 1990 to 2019. AAPC, Average annual percentage changes.

At the national level, the most pronounced increase in the prevalence of PVF between 1990 and 2019 was in Libya (AAPC 6.8143 (6.6194 to 7.0113)). The most pronounced increase in the prevalence of PVOM between 1990 and 2019 was in Libya (AAPC 3.7554 (3.6095 to 3.8991)). The most pronounced increase in the prevalence of PVSO between 1990 and 2019 was in Libya (AAPC 3.5391 (3.4083 to 3.655)). The most pronounced increase in the prevalence of SV between 1990 and 2019 was in Oman (AAPC 0.4561 (0.4338 to 0.478)). The most pronounced decrease in the prevalence of PVF between 1990 and 2019 was in Thailand (AAPC -2.4031 (−2.4634 to −2.3328)) (Figure 2 and Supplementary Table S1).

**Figure 3:**
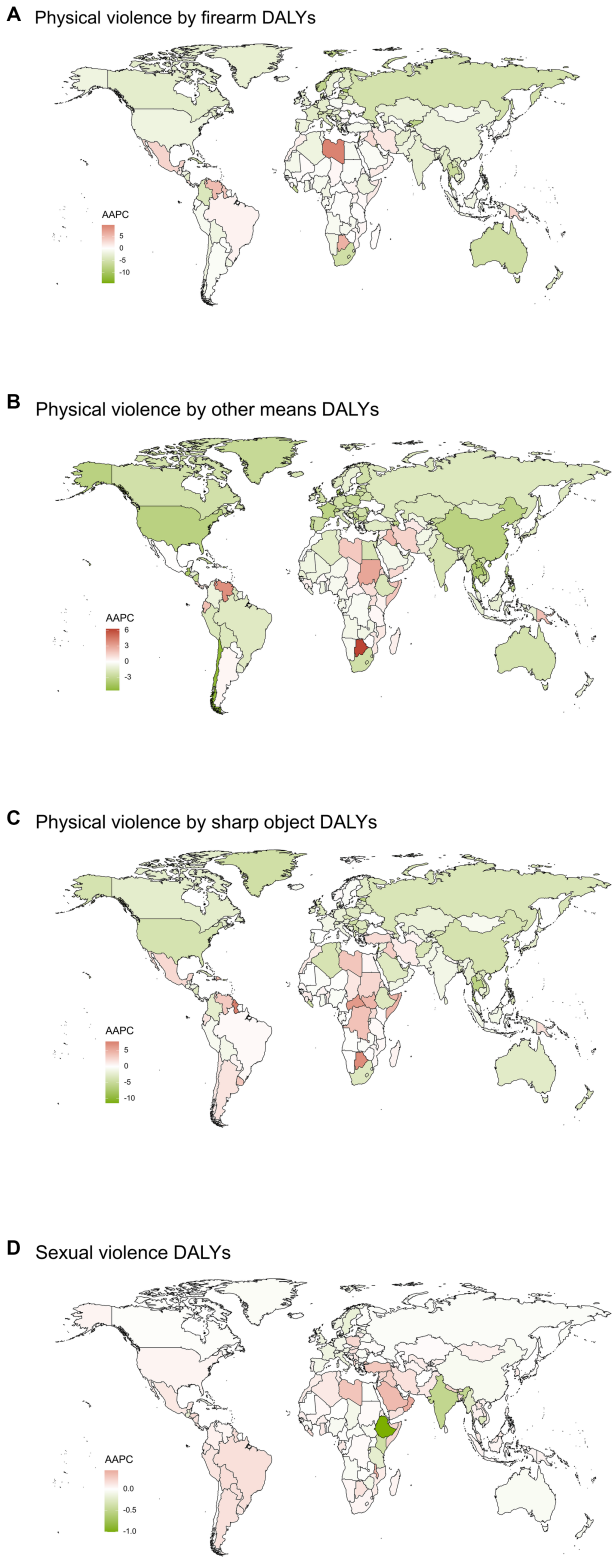
Global map of average annual percentage changes in DALYs of Physical violence by firearm **(A)**, Physical violence by other means **(B)**, Physical violence by sharp object **(C)** and Sexual violence **(D)** from 1990 to 2019. DALYs, Disability-adjusted life years; AAPC, Average annual percentage changes.

Between 1990 and 2019, the largest increase in DALYs from PVF was observed in Libya [AAPC 9.5548 (6.7469 to 15.5934)]. Between 1990 and 2019, the largest increase in DALYs from PVOM was observed in Botswana [AAPC 6.2725 (6.0951 to 6.4082)]. Between 1990 and 2019, the largest increase in DALYs from PVSO was observed in Guyana (AAPC 7.7256 (6.3895 to 9.1994)). Between 1990 and 2019, the largest increase in DALYs from SV was observed in Oman [AAPC 0.4425 (0.4206 to 0.464) (Figure 3 and Supplementary Table S1)].

## Discussion

### Background

To our knowledge, this is the first study to describe prevalence and rates of change of PVF, PVOM, PVSO, and SV among young women aged 10–24 years, from 204 countries, from 1990 to 2019, at the global, regional, and national levels. It uses a broad range of data sources including population-based surveys and administrative records to estimate national trends, which were aggregated to generate regional and global figures. The inclusion of multiple datasets helps to reduce biases that could arise from any single source. The long times pan of three decades also allows for the assessment of meaningful changes over time.

### Global trends

The results of this study indicated that the global prevalence of PVOM, PVSO and SV decreased between 1990 and 2019. These decreasing trends may be attributable to increased awareness of these public health issues, policy and programs on preventing violence against women in recent decades (Correa et al., 2009; Labronici et al., 2010). As discussed in a previous systematic review, interventions aimed at challenging rape myths and stereotypes have been shown to be effective in reducing acceptance of such attitudes (Hudspith et al., 2023). However, the global SV prevalence decreased slightly between 1990 and 2019, but increased slightly between 2010 and 2019, which warrants further investigation. Previous study found that prevalent myths surrounding women’s conduct during intimate partner rape greatly impact societal attitudes (Lilley et al., 2023). The increasing PVF prevalence in recent years could be due to increased disclosure of such experiences or due to setbacks in global progress, or a combination. Notably, PVF prevalence increased in low-SDI countries between 2010 to 2019, indicating that women in poorer, less developed countries may be particularly vulnerable. The increase in PVF was also more pronounced in younger age groups (10–14 and 15–19 years), suggesting the need to better tailor prevention efforts to adolescents and young people worldwide.

### Trends across age groups

Among the different age groups, Young women aged 10–14 years were the age group with the greatest decline in PVSO prevalence between 1990 and 2019.The larger declines among younger age groups are encouraging and suggest that preventive interventions tailored for adolescents and youth can be effective. However, the prevalence of all four types of violence against women in 2019 remained high in these age groups, indicating the need for further intensified actions. For adolescents and youth, exposure to violence at a young age can have severe and long-lasting impacts on their development and health (Dahlberg and Krug, 2006; McDonald and Merrick, 2013). Due to their life stage, younger women may also face distinct vulnerabilities to violence. For instance, adolescent girls are more prone to experience violence from intimate partners, family members and peers (Greenfield, 1997; Kaukinen and DeMaris, 2005). They also encounter more barriers to disclosing and seeking help from violence (Sabri et al., 2015). Therefore, tailored interventions for this population should address their specific needs and vulnerabilities, such as increasing the minimum age of marriage, improving mandatory reporting of violence against minors, and integrating prevention of violence against women and girls into education curricula at an early stage. Ni3: VRA outcome measure could help evaluate the effectiveness of such tailored interventions (Debowska et al., 2019).

### Trends by SDI quintile

The trends in the prevalence of PVF, PVOM, PVSO and SV varied according to SDI quintile. The Low SDI countries had the largest increase in PVF prevalence among young women. The decreasing trend was more pronounced in high and high-middle SDI countries, while the PVF prevalence actually increased in low SDI countries. In higher SDI countries, greater political will, policy commitments and availability of resources for preventing violence against women in recent decades likely contributed to the larger declines. The SV prevalence of High SDI was the only one that rose among the different SDIs in SV between 1990 and 2019. indicating that there is still a lot of work to be done in high SDI countries to eliminate these prevalent problems. For low-SDI countries, limited resources, lack of policy/legal frameworks, and deeply entrenched gender inequalities pose greater challenges to overcoming violence against women (Halkos and Gkampoura, 2021).

### Regional trends

Trends in the prevalence of PVF, PVOM, PVSO and SV also varied in different regions. Regionally, between 1990 and 2019, young women in Andean Latin America has the largest increase in PVF prevalence, and Oceania has the largest increase in PVOM, PVSO prevalence and PVOM, PVF DALYs. Caribbean has the largest increase in SV prevalence. The decreasing trends were more pronounced in East Asia, South Asia and tropical Latin America. In contrast, the increasing trends were more pronounced in Oceania and the Caribbean. In East Asia and South Asia, greater economic growth, public investments in healthcare and education as well as policy/legal reforms supporting women’s rights and gender equity over past decades may have contributed to the larger declining trends (Jeyaseelan et al., 2007). The increasing trends in Oceania and Caribbean highlight the need for concern. Possible drivers include poverty, income inequality, lack of women’s empowerment and entrenched gender attitudes accepting of violence - all of which can fuel the vulnerabilities of violence in these regions (DeShong and Haynes, 2016). Urgent policy, advocacy and prevention efforts are needed to reverse the worsening situation, with support from the international community.

### Limitations

There are several limitations to consider. First, the quality of input data and methods used for estimation can vary between sources, which may introduce uncertainties in the final aggregated figures. Not all world regions had sufficient data to be included, especially for trends analysis. Under-reporting is also a concern given the sensitive nature of violence against women. Second, the definitions and measurements of different types of violence were not standardized across all data sources, although efforts were made to categorize them broadly. This can impact the validity of aggregating results. Third, regional groupings are very broad and heterogeneous. Significant variations are masked within each region. Socio-demographic factors like age, income, education and marital status also shape risks and impacts but were not extensively explored. Finally, the findings show correlation but do not prove causation. While decreased prevalence over time suggests the effects of prevention efforts and social changes, a variety of complex contextual factors are involved. Reverse causality is also plausible with lower prevalence driving policy/social responses.

### Directions for future research

Further exploratory work should compare exemplary and worsening jurisdictions to discern transferable lessons, examine underlying socioeconomic and cultural determinants through both quantitative and qualitative lenses, and longitudinally assess long-term impacts of early exposure. Continued collection of high-quality, standardized data and rigorous evaluation of comprehensive national programs and grassroots projects can establish evidence-based practices for Extension. Concerted global efforts are imperative to build on progress and overcome remaining challenges.

## Conclusion

In summary, this study provides a valuable overview of global violence against women trends and highlights regions that have made progress as well as those needing urgent action. However, the results should be interpreted with consideration of methodological limitations and the nuances that exist within summarized figures. Continued collection of high-quality, standardized data and in-depth analyses of country contexts are needed to inform evidence-based solutions to this critical issue.

## Data availability statement

All data used in this study can be freely accessed at the Global Burden of Disease 2019 portal (https://vizhub.healthdata.org/gbd-results/).

## Ethics statement

Approval for the research protocol of the GBD study has been granted by the Institutional Review Board at the University of Washington. The GBD inquiry will be conducted in strict conformity with the policies and procedures established by the University of Washington, and in accordance with relevant federal, state, and local statutes.

## Author contributions

YC and HL conceived the presented idea. HL, YC, and DW performed the manuscript writing. YC, GW, and PD were involved in the acquisition and processing of data. YC, HL, GW and DW was involved in the interpretation of data. All authors contributed to the article and approved the submitted version.
